# Screw sense and screw sensibility: communicating information by conformational switching in helical oligomers

**DOI:** 10.1039/d2cs00982j

**Published:** 2023-03-16

**Authors:** David T. J. Morris, Jonathan Clayden

**Affiliations:** a School of Chemistry, University of Bristol, Cantock's Close Bristol BS8 1TS UK j.clayden@bristol.ac.uk

## Abstract

Biological systems have evolved a number of different strategies to communicate information on the molecular scale. Among these, the propagation of conformational change is among the most important, being the means by which G-protein coupled receptors (GPCRs) use extracellular signals to modulate intracellular processes, and the way that opsin proteins translate light signals into nerve impulses. The developing field of foldamer chemistry has allowed chemists to employ conformationally well-defined synthetic structures likewise to mediate information transfer, making use of mechanisms that are not found in biological contexts. In this review, we discuss the use of switchable screw-sense preference as a communication mechanism. We discuss the requirements for functional communication devices, and show how dynamic helical foldamers derived from the achiral monomers such as α-aminoisobutyric acid (Aib) and *meso*-cyclohexane-1,2-diamine fulfil them by communicating information in the form of switchable screw-sense preference. We describe the various stimuli that can be used to switch screw sense, and explore the way that propagation of the resulting conformational preference in a well-defined helical molecule allows screw sense to control chemical events remote from a source of information. We describe the operation of these conformational switches in the membrane phase, and outline the progress that has been made towards using conformational switching to communicate between the exterior and interior of a phospholipid vesicle.

## Abstract 2

Helical oligomers devoid of configurational chirality can communicate information when an externally induced screw-sense preference propagates through the chain.

## Graphical abstract

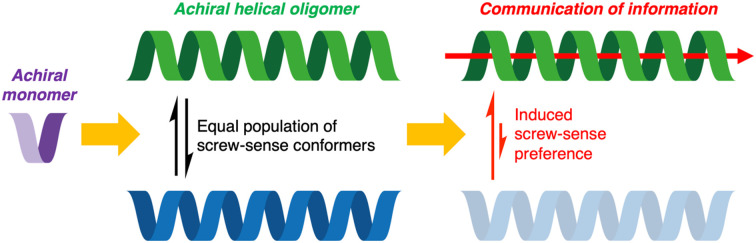

## Introduction

**Fig. 1:**
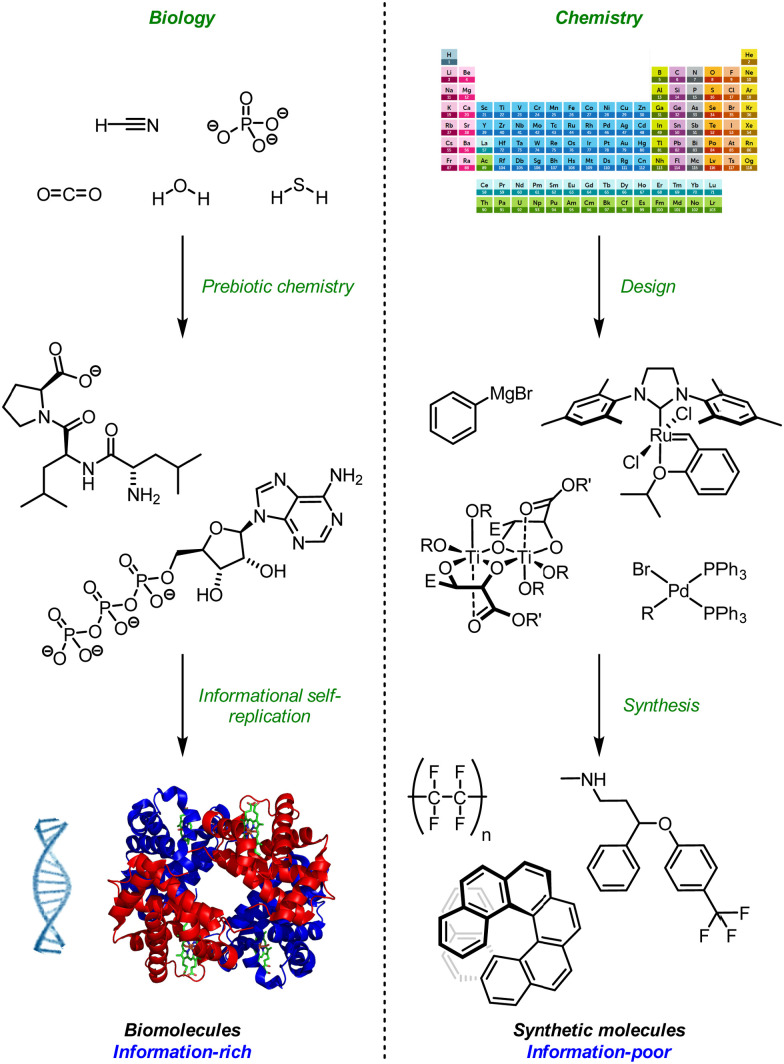
Information and design in Biology and Chemistry. Adapted from ref. 14–16 with permission from the Royal Society of Chemistry, 2020; Wikimedia commons, 2020; and the American Association for the Advancement of Science, 2020.

Biology and organic chemistry share the same vocabulary of carbon-based molecular structure, but differ in the complexity of their syntax. Chemistry classically deals with well-defined single compounds, with crystallisation and crystal structure determination still representing in many areas the pinnacle of purification and molecular characterisation. In contrast, biology emerges from complex out-of-equilibrium compartmentalised dynamic mixtures.^1^ Biological systems are necessarily information-rich; chemical systems are classically information-poor (Fig. 1).^2,3^ Biology possesses sophisticated, evolved mechanisms for the storage, duplication, amplification, communication and processing of information.^4–6^ Chemistry has only just begun to explore the possibilities offered by building designed structures that mimic these information-processing abilities. Indeed, advances have recently been made in self-replication,^7–9^ alternative genetic codes,^10^ and translation machines.^11,12^ In this review, we will summarise progress towards the design of a purely synthetic mechanism for the communication of information by means of molecular conformation. We will describe fundamental requirements of molecular communication devices, and show how dynamic helical foldamers that may be switched between their left- and right-handed screw-sense conformations fulfil these requirements. We will outline the difficulties that need to be overcome in designing and synthesising such screw sense-convertible foldamers, and show that certain classes of oligomers seem uniquely suited to the purpose.

## Requirements of molecular communication devices

Inspired by the work of Boltzmann and Gibbs on statistical thermodynamics in the late 19th century, Claude Shannon proposed in 1948 a ‘theory of information’ detailing the function of a general communication system consisting of an information source, a transmitter, a signal, a communication channel (where the signal may be subject to noise or distortion) and receiver components.^13^ While this original treatment was applied to electrical engineering, and is indeed viewed as underpinning the information technology revolution of the early 21st century, it is clear that the theory has much more general applicability.^17,18^

Each of the principal components in Shannon's theory can take molecular manifestations. Biology has evolved molecular mechanisms that allow complex instructions to be relayed on a variety of scales with negligible signal loss. For example, the membrane-bound receptor rhodopsin enables transmembrane communication and phototransduction.^19^ In ‘Shannonian’ terms, rhodopsin's cofactor (retinal) transmits information (the incidence of light of a suitable wavelength) through the rhodopsin ‘channel’, which is then ‘received’ by the corresponding signalling protein, transducin. Numerous chemical subtleties likewise make G protein-coupled receptors (GPCRs) highly effective communication devices.^20^ The identification of these subtleties^21–23^ has allowed chemists to use concepts from evolved natural systems to help inform the design of artificial communication devices.

The most pertinent requirement for the design of a molecular communication device is that the medium through which the information is communicated (the channel) must be able to adopt more than one state, but not significantly more than two. A single-state device cannot respond to the input of information; conversely having too many states requires an unfeasible degree of complexity in the receiver if it is to distinguish them reliably.

Molecular communication devices typically operate under thermodynamic control (ratcheted devices are an exception^24–26^): the states of the channel must be local energy minima. These states must be sufficiently similar in energy that their relative population can be biased in response to a stimulus (informational input). The channel must also be dynamic: the minima must be separated by a barrier sufficiently low for communication to occur on practically useful timescales. If these criteria are not satisfied, then the stimulus will not be able to induce communication through the channel, either because the induced state is too high in energy, or because the barrier for interconversion of states is too high.

Dynamic chirality forms an ideal conceptual framework for devising mechanisms of molecular communication. Two enantiomers represent two states, and the enantiomers of a chiral structure are necessarily identical in energy in an achiral environment. For enantiomers that can interconvert through a low enough barrier, their equilibrium may be biased by a chiral signal to favour one of the enantiomeric states, which become diastereoisomeric, and hence energetically unequal under the chiral influence.

Dynamic molecules that racemise or epimerise on an appropriate timescale are numerous: these processes include pyramidal inversion in chiral amines^27^ or phosphines,^28^ ring-flipping in *meso* cyclohexanes,^29^ and atropisomerism (or near-atropisomerism) in biaryls or heterobiaryls.^30,31^ However, to communicate information over long distances, molecules must contain more than just a single, localised stereogenic centre or axis, and must adopt a single globally defined chiral conformation.

One class of chiral structure fits this requirement uniquely: the helix. Helices are chiral by nature – they twist anticlockwise (to the left, *M*) or clockwise (to the right, *P*) as they propagate along an axis. They also have translational symmetry – in other words they possess a form of ‘linear chirality’, in which the same dissymmetry is expressed uniformly along the length of the helix. A helix which is left-handed at one location is left-handed everywhere, and this makes helices ideal as communication devices for chiral messages.

**Fig. 2:**
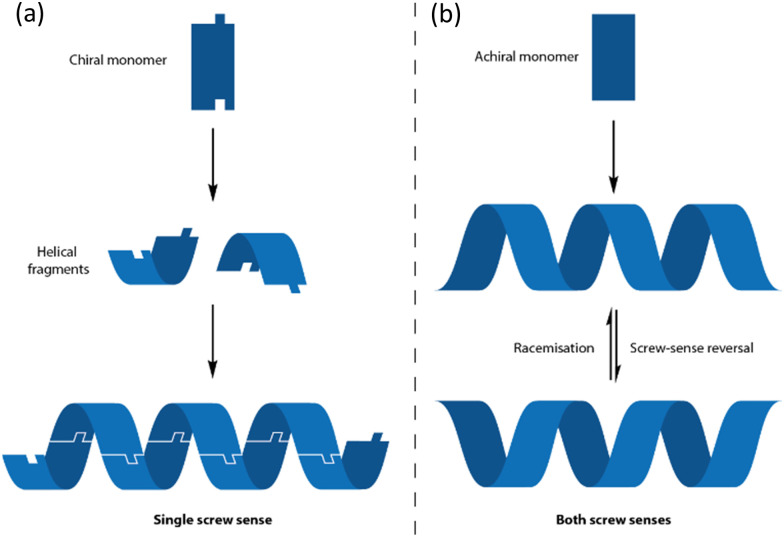
The formation of helices from (a) chiral and (b) achiral monomers.

**Fig. 3:**
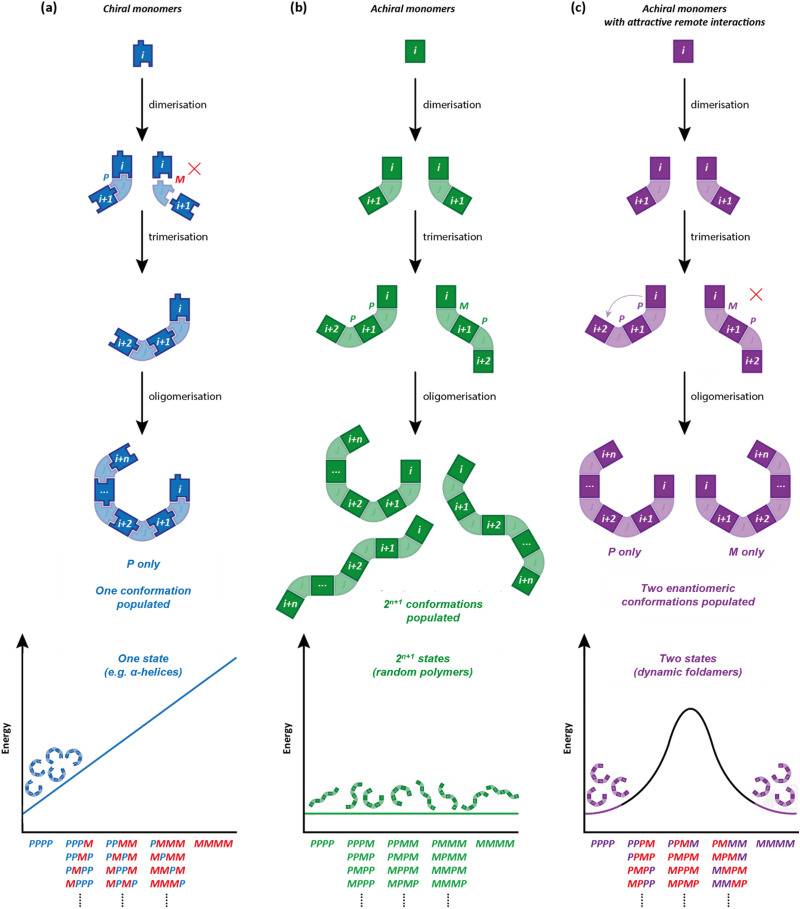
Global conformations in oligomers comprised of (a) chiral monomers (b) achiral monomers with no long-range interactions and (c) achiral monomers with attractive *i* to *i* + 2 interactions.

Helical structures have the further advantage of being commonly adopted by polymeric molecules, both natural and synthetic – not least for the simple mathematical reason that a helix necessarily arises from the concatenation of identical three-dimensional objects where each is related to its neighbours by a consistent vector. As an example, oligopeptides of l-amino acids almost invariably^32^ adopt right-handed (*P*) α-helical structures. But this type of helix is not useful as a communication channel of the type outlined above: due to the central chirality in the amino acid residues, *P* and *M* α-helices of this type are diastereoisomeric conformers. These conformers differ substantially in energy (*P* helices being 21 kJ mol^−1^ more stable than *M* helices in simulated neutral α-helical Ala_6_) and so *M* α-helices are not observed at equilibrium.^33^ Any attempt to bias this equilibrium would need to overcome this substantial penalty (Fig. 2a and 3a).

Removing the chiral centres from a peptide solves this problem: a helix of achiral monomers can feasibly adopt either a left- or a right-handed structure (Fig. 2b).^34^ Conceptual poly-demethylation of polyalanine to form polyglycine is unfruitful in practice: polyglycine is conformationally promiscuous^35^ and forms insoluble aggregates.^36^ But conceptual poly-methylation of polyalanine gives a polymer of the achiral α,α-disubstituted amino acid aminoisobutyric acid (Aib).

Poly-Aib suits perfectly as an achiral polymer that adopts a chiral ground-state conformation.^34^ It is well-established that Aib oligomers are helical – in fact they adopt not an α-helical, but a 3_10_-helical structure,^37^ with exactly 3 residues per turn, built up from ten-membered hydrogen-bonded rings between amino acid *i* and amino acid *i* + 3. This remote interaction between non-adjacent monomers in the oligomer chain is important for a rather subtle reason, and one that makes many alternative and apparently helical oligomers of achiral monomers unsuitable as conformational communication devices. Before exploring poly-Aib in more detail, it is worth exploring first why it functions so well as a conformationally responsive structure, in the way that some urea-linked oligomers of *meta*-phenylenediamine, which we have also explored in this context, did not.^38^

An oligomeric structure in which a helical global conformation emerges from the interaction of an ensemble of achiral monomers suffers from a problem that does not arise when chiral monomers are concatenated into a helix. As noted in Fig. 2, a helical polymer of enantiopure chiral monomers will always have a preferred screw sense, as a twist to the left or to the right requires alternative diastereoisomeric, and hence energetically inequivalent, interactions between monomers (Fig. 3a). But with achiral monomers, the junction between adjacent monomers can necessarily adopt two enantiomeric conformations, which must be equal in energy. In the absence of more remote interactions within the extended oligomer, the consequence would be that the oligomers populate all isoenergetic permutations of *P* and *M* monomer junctions in equal measure, making the oligomer a random coil (Fig. 3b). This conformational promiscuity can in fact give rise to valuable properties and has been exploited in materials polymers^39^ and hydrogels,^40^ but it does not lend itself to the communication of information.

**Fig. 4:**
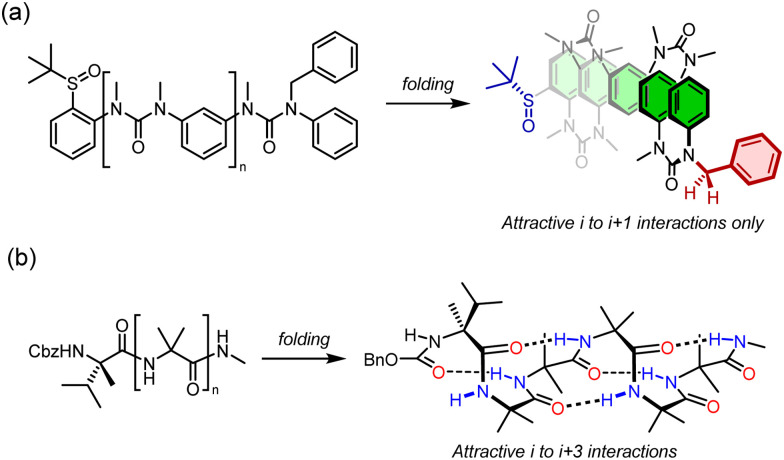
Inter-monomer interactions in configurationally achiral helices. (a) Breakdown of helicity in *meta*-phenylenediamine oligoureas (*M* helix depicted). (b) Persistent helicity in Aib oligomers (*P* helix depicted).

**Fig. 5:**
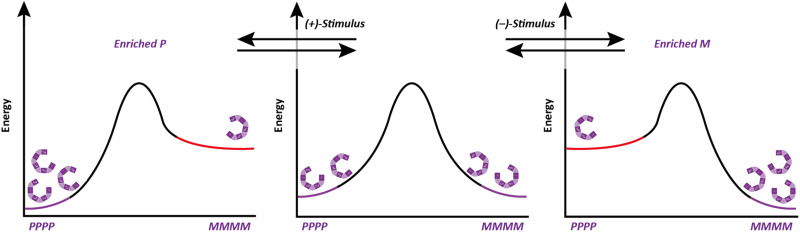
Symmetry-breaking in configurationally achiral oligomers.

Importantly, the situation is different again if a monomer experiences an interaction – either attractive or repulsive – with more remote components of the oligomer. In such a case, a dramatically reduced degree of conformational promiscuity emerges. Fig. 3c illustrates the outcome if monomers *i* and *i* + 2 experience an attractive interaction: adjacent junctions having the same direction of twist are favoured, and in the ideal case a uniform helix again emerges, but without a preference for overall *M* or *P* screw sense. Such conformations arise from the *i* to *i* + 2 interactions in Suginome's poly(quinoxaline-2,3-diyl)s,^41^ the attractive π-stacking interactions in Huc's helical oligomeric aromatic amides,^42–45^ the attractive *i* to *i* + 3 hydrogen-bonding interactions in Aib oligomers,^46^ as well as in polymers organised by dipole–dipole or π–π-stacking interactions such as polyisocyanates and polyacetylenes.††We also reported some oligo(xanthene-1,8-dicarboxamide) structures in which dipolar repulsion between the amides induced a single global conformation in extended structures where adjacent axes were forced to adopt the opposite handedness to each other (repulsive *I* to *i* + 1 interactions).^123^ This translated throughout the whole structure, with each pair of axes exhibiting exclusively oppositely oriented amides. By introducing a symmetry-breaking chiral influence on one end, it was possible to induce a global conformational preference in favour of one of the pseudoenantiomers. A Grignard addition was performed at different points along the oligomeric structure giving up to 95% diastereoselectivity as a result of the remote chiral information.^47^ The strength of these inter-monomer interactions dictates the gradient of the resulting potential energy surfaces. The two limiting outcomes that arise (Fig. 3b and c) are illustrated by two families of achiral helices explored in the last 20 years: *meta*-phenylenediamine oligoureas (Fig. 4a)^38,48^ and oligomers of Aib (Fig. 4b).^46^ The former are poorly controlled and adopt a widely populated conformational landscape, principally because each aromatic ring interacts solely with its immediate neighbour, and the conformation of one monomer has no bearing on the conformational preference of its neighbours. Oligomeric aromatic ureas had only limited success in transmitting stereochemical information. Aib oligomers are very different. Every monomer is part of three different chiral hydrogen-bonded rings, all of which must adopt the same absolute configuration. Thus, a chain of Aib residues can adopts only an all-*M* or an all-*P* conformation (and indeed we have managed to quantify with high accuracy the degree to which this holds true).^49^ Moreover, Aib oligomers interconvert between these two enantiomeric conformations on the millisecond timescale (Δ*G*^‡^_296 K_ = 35 kJ mol^−1^ in CD_3_OD for a configurationally achiral Aib nonamer). As the two conformations populated by an oligo-Aib chain are enantiomers, they will each interact differently with a chiral stimulus differently (Fig. 5). The potential energy surface experienced by the achiral oligomer is desymmetrised, and one of the two enantiomeric conformations is favoured over the other. ^13^C NMR (or other nuclei) spectroscopy can be used to calculate the degree of screw-sense preference or ‘helical excess’ at any point in the helix by examining the anisochronicity (chemical shift separation) between pairs of diastereotopic methyl groups in any residue of an Aib helix. A local chiral stimulus can thus be used as a local informational input with a global conformational consequence, allowing the helix to provide a mechanism for molecular communication.

**Fig. 6:**
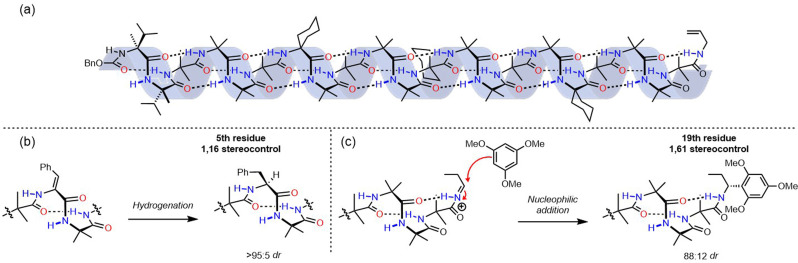
(a) 1,61 control of screw-sense preference. (b) Asymmetric induction in the reduction of didehydrophenylalanine and (c) nucleophilic addition to an acyliminium ion.

An induced screw-sense preference can lead to control of chemical function. When an Aib helix is terminated with two contiguous chiral l-α-methylvaline residues (at the N terminus in Fig. 6a), an Aib unit five residues away experiences a *P *:* M* helical ratio of 96 : 4 at 313 K in THF, which improves to 99 : 1 upon cooling to 223 K. We suspected that the screw-sense preference induced in the helix could be used to effect a diastereoselective reaction.^50^ A variety of foldamers were prepared with reactive prochiral functionality replacing certain residues of the scaffold depicted in Fig. 6a to investigate remotely controlled diastereoselective reactions. The fifth residue was replaced with a *Z*-didehydrophenylalanine unit, which was hydrogenated using an achiral catalyst (Fig. 6b). This reaction resulted in formation of the diastereomer containing the corresponding l-phenylalanine with >95 : 5 diastereoselectivity – a reaction in which the face of attack on an alkene is controlled by a remote stereogenic centre, with the control mediated by the induction of screw-sense preference.^51^

This concept was extended to an oligopeptide in which 19 achiral amino acids, and hence six turns of a 3_10_ helix, separated an electrophilic acyliminium ion from the nearest chiral centre. The screw-sense induction imposed by an N-terminal pair of l-α-methylvaline units gave rise to an 88 : 12 diastereomeric ratio of adducts when 1,3,5-trimethoxybenzene was used as a nucleophile. This result demonstrates that screw-sense preference in Aib-derived helices has the potential to communicate stereochemical information over distances of 4 nm in solution, and can even control asymmetric chemical reactions over these distances. Aib-derived foldamers satisfy the necessary requirements for the communication of information over long distances, and can process information by performing a chemical function. However, in these examples the source of chiral information is inherent to the molecule itself – in none of these examples is the oligomer able to read information. Higher functions in molecular communication devices require the point of informational input in the device to be able to adopt different states, and to communicate its state to the communication channel.

## Screw-sense switching in helical oligomers

Stimulus-responsive control of molecular function lies at the core of every significant biological process, and ways to control the conformational dynamics of synthetic molecules is an attractive prospect for the design of new functional molecular systems. Foldamers can be engineered to respond to stimuli by incorporation of stimulus-sensitive functionality, which has been used for advances in host–guest chemistries, biomimicry and medicine.^52–54^ Using control of screw-sense to achieve molecular function is a well-established field^55–58^ that has the potential to provide new molecular communication devices.

**Fig. 7:**
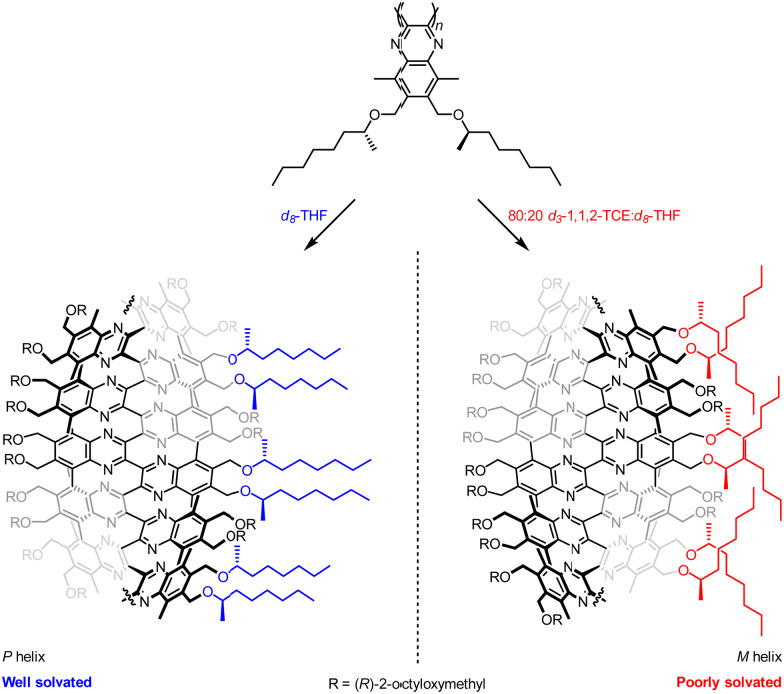
Suginome's screw-sense switching in poly(quinoxaline-2,3-diyl)s.

Suginome and co-workers have explored the screw-sense switching of polymeric helices derived from poly(quinoxaline-2,3-diyl)s (PQXs).^59^ Stereogenic centres located within the polymer can induce a screw-sense preference, but the influence of this central chirality on the conformation of the polymer is modulated by external stimuli, (Fig. 7) especially as a consequence of solvation in even only subtly different solvents.

Small-angle neutron scattering (SANS) experiments on 100 mer PQXs bearing (*R*)-2-octyloxymethyl substituents revealed a dramatic dependence of screw-sense preference on solvent.^60^ Polymers in *d*_8_-THF (*P* helix) and in 80 : 20 *d*_3_-1,1,2-TCE : *d*_8_-THF mixture (*M* helix) exhibited equal and opposite Cotton effects in their circular dichroism (CD) spectra, indicating a quantitative inversion of screw-sense preference.

With the aid of computational simulation, the SANS data recorded in these mixtures indicated that THF solvates the (*R*)-2-octyloxymethyl groups well, allowing them to adopt an approximately linear conformation extending out into the solvent, and orienting the quinoxaline-2,3-diyl units in the main chain so they adopted a *P*-helical conformation.

In contrast, an 80 : 20 1,1,2-TCE : THF mixture induced a more compact conformation in the (*R*)-2-octyloxymethyl groups in order to mitigate their interactions with solvent and maximise inter-chain van der Waals interactions. This compacted conformation reoriented the quinoxaline-2,3-diyl units in the main chain into an *M*-helical conformation, and solvation-dependent screw-sense preference was used to modulate the conformation of PQXs bearing a variety of chiral groups by exploiting very subtle solvent effects. For example, substituted lactate side chains resulted in a switch in screw-sense preference even between similar ethereal solvents 1,2-dimethoxyethane and *tert*-butyl methyl ether.^61^ Judicious selection of chiral substituents led to PQXs that could discriminate between a single methylene unit in the solvent, giving rise to a screw-sense preference switch between *n*-heptane and *n*-octane.^62^ Screw-sense preference was also induced by using configurationally achiral PQXs in the chiral solvent limonene.^63^

Interestingly, the intrinsic screw-sense preference can also be modulated by the degree of polymerisation of the PQX.^64^ The PQXs are synthesised by living polymerisation of the corresponding 1,2-diisocyanobenzenes, so interspersing selected functional groups among the monomers provides helical polymers with specific properties. For example, PQXs exhibiting circularly polarised luminescence were generated by including terphenyls within the polymer.^65^ Catalytically active units embedded in the helical polymer gives rise to asymmetric reactivity. Thus PQXs decorated with triarylphosphines catalyse atroposelective Suzuki–Miyaura couplings,^61^ those with oxazaborolidines catalyse enantioselective C–C bond cleavage,^66^ and those with N-heterocyclic carbenes catalyse enantioselective cyclopropanation of olefins.^67^

Helical poly(acetylene)s adorned with axially chiral 2,2′-bis(methoxymethoxy)biphenyls display helical chirality. Maeda and co-workers discovered that the screw-sense can be quantitatively induced by treatment with chiral secondary alcohols.^68^ This process is reversible, allowing either screw sense to be adopted. A chiral memory effect results in retention of screw-sense preference even after the chiral secondary alcohol is removed. Furthermore, the insolubility of the polymer permits its use as a switchable chiral stationary phase for the separation of enantiomers. The order of elution of the analyte's enantiomers may be switched by prior enantioenrichment of the helix using different chiral alcohols.

**Fig. 8:**
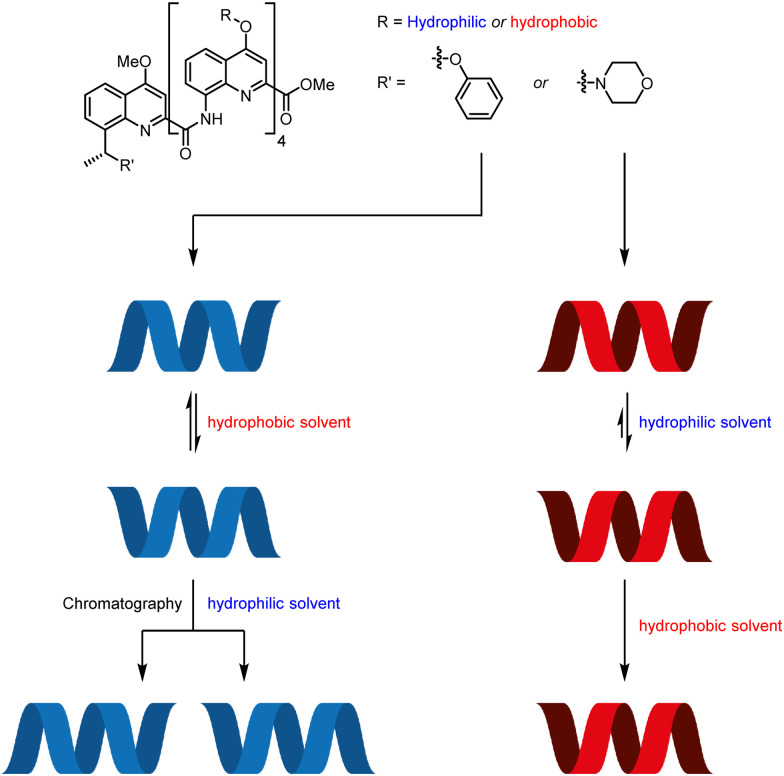
Screw-sense induction in Huc's aromatic oligoamide foldamers.

The aromatic oligoamide foldamers developed by Huc and co-workers are helical oligomers that engage in a variety of host–guest interactions.^69–71^ Monomers bearing no chiral centres form racemic helices, lending themselves to screw-sense induction by external stimuli. These racemic helices interconvert through a barrier that varies with oligomer length and with solvent.^72,73^ This feature was exploited by using a chiral group to promote one screw sense in a hydrophilic foldamer in organic solvent, where helical interconversion was fast, then kinetically trapping that screw sense by dissolving the foldamer in water, where helical interconversion was slow (Fig. 8).^74^ A similar principle operates in hydrophobic foldamers in other solvents.

A hydrophilic octamer with a C-terminal l-phenylalanine residue was prepared by solid-phase synthesis, giving a mixture of diastereoisomeric *P* and *M* helices. The helices could be separated by reverse-phase HPLC, indicating slow helical inversion in aqueous media. The separated helices persisted for five days in D_2_O, at which point no equilibration was observed. However, in *d*_6_-DMSO, conformational equilibrium was reached within 2 days, converting either sample into a 70 : 30 mixture of *P *:* M* helices, according to ^1^H NMR and CD spectroscopy. Although the diastereomeric helices were separable, it was also desirable to use this solvent-dependent helical inversion to obtain diastereomerically pure helices. The chiral screw-sense inducer was thus brought closer to the C-terminal quinoline by appending chiral benzylic centres to the 8-position of the quinoline.

Hydrophobic pentamers with enantioenriched benzylic morpholines and phenyl ethers at the C terminus were synthesised. (*R*)-Phenyl ethers induced no screw-sense preference, but an (*R*)-benzylic morpholine substituent gave rise to an almost exclusive preference for *M* helicity in 3 : 1 CD_3_CN : CDCl_3_, *d*_3_-MeOH and *d*_6_-DMSO. This was increased to complete *M*-selectivity by protonation of the quinolines. Quantitative screw-sense induction also arose in hydrophilic pentamers in water. These examples make it clear that environment can alter the dynamics of screw-sense reversal, and may become key to the functioning of molecular communication devices.

**Fig. 9:**
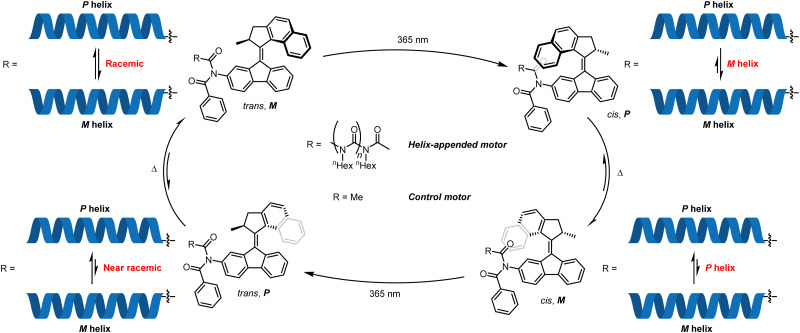
Feringa's screw-sense switching by modulation of the influence of an overcrowded alkene-based molecular motor.

Conformational switching induced from a single localised controller has been reported by Feringa and co-workers,^75–77^ who described a three-state switch of screw-sense preference in a configurationally achiral polyisocyanate helix.^78^ By means of an enantioenriched overcrowded alkene motor, the influence of the local axial chirality in the motor on the screw-sense preference of the polyisocyanate helix could be modulated to induce *P*, *M* and racemic conformations in the helical polymer. The motor functions by iterative photochemical alkene isomerisations that give metastable states exhibiting a certain local helical chirality, which relax to an alkene with the opposite local helical chirality. When a naphthalene unit protrudes towards an appended poly-(*n*)-hexylisocyanate, the motor induces a screw-sense preference (Fig. 9). When the naphthalene protrudes away from the polyisocyanate (*trans* olefin), its influence on the polyisocyanate is greatly reduced, giving rise to a helically racemic polyisocyanate, as inferred by CD spectroscopy.

To quantify the CD contributions from the helix itself, a polymer-free motor was synthesised, and its CD spectrum subtracted from that of the polymer-appended motor. In the *trans* form (Fig. 9, top left), the motor with and without an appended polymer exhibited near identical CD spectra, indicating that the polyisocyanate helix was conformationally racemic. Irradiation (*λ* = 365 nm) at −20 °C gave a *cis* alkene with a metastable local *P* configuration (Fig. 9, top right). At this point, the control motor and the polymer-appended motor differed in their CD spectra, indicating induction by the motor of a preferred *M* screw sense in the polyisocyanate. Thermal reversion over 30 minutes at ambient temperature to a thermodynamically stable *cis* alkene with local *M* configuration (Fig. 9, bottom right) inverted CD spectra for both the motor alone and the polymer-appended motor, showing that the polyisocyanate now adopts a *P* helical conformation. Photoisomerisation back to the *trans* alkene removed the asymmetric influence of the naphthalene unit on the polyisocyanate (Fig. 9, bottom left), substantially reducing the conformational preference of the polyisocyanate.

This work was extended to the control of metal-coordinating double helices by appendage of an oligo(2,2′-bipyridine) onto each half of a *C*_2_-symmetrical overcrowded alkene-based molecular motor.^79^ In the *cis* forms, either *P* or *M* double helical Cu(i) complexes formed depending on the helical chirality in the motor. In the *trans* forms, intramolecular complexes are impossible, and strands from different molecules became interwoven to form aggregates. This example again constitutes a three-state switch where screw-sense preference could be selected, but additionally, one of the states triggers a reversible supramolecular polymerisation event.

**Fig. 10:**
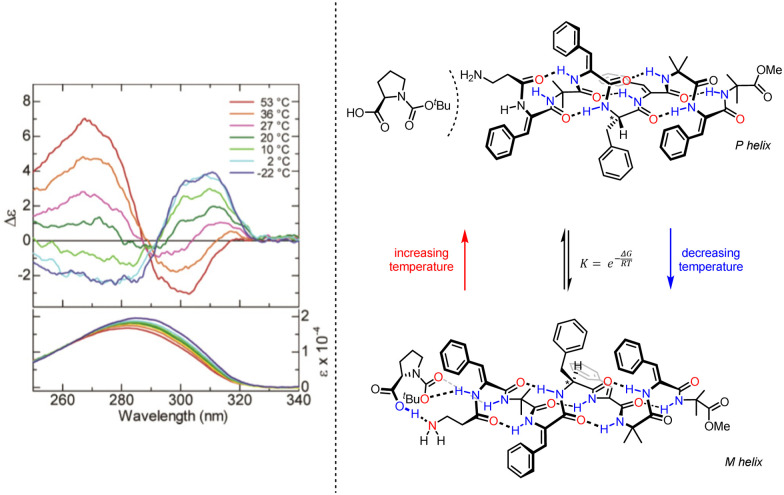
Concentration and temperature-dependent screw-sense switching in 3_10_-helical oligopeptides. Reprinted (adapted) with permission from ref. 83. Copyright 2020 American Chemical Society.

Inai and co-workers have investigated the chiroptical properties of helices containing a mixture of chiral and achiral monomers.^80–82^ An otherwise configurationally achiral 3_10_-helical oligomer with an embedded l-phenylalanine residue in provided stimulus-responsive control of screw-sense preference.^83^ The embedded l-phenylalanine was sufficient to impart a screw-sense preference to the whole helix, which augmented with decreasing temperature. The screw-sense preference of the helix could however be inverted by binding a Boc-d-proline ligand non-covalently at the N terminus of the helix, with the influence of the ligand on the helix conformation modulated by altering the concentration and temperature of the system (Fig. 10).

## Helical oligomers as communication channels

Numerous cases of screw-sense control in covalent polymers have been described, but there are also many examples of screw-sense control in helical supramolecular polymers^84–88^ and liquid crystals.^89,90^ Polymers can be prepared with different lengths and radii, but more crucially with different properties, functions and sensitivities to stimuli. However, the screw-sense induction in these examples arises from global conformational influences distributed through the length of the polymer. The communication of information necessitates localised, spatially defined inputs and outputs, separated by a ‘communication channel’.

The first example of informational communication by spatial translation of screw-sense preference was published by Inai and co-workers, who extended their work on the use of Boc-d-proline to control screw-sense preference in 3_10_-helical oligopeptides to form a simple molecular communication device.^91^ A configurationally achiral heptadecapeptide comprised of Aib and *Z*-α,β-dehydrophenylalanine was synthesised, with the 14th and 16th residues replaced with (*Z*)-β-(4,4′-biphenyl)-α,β-didehydroalanine as a diagnostic spectroscopic reporter with a distinctive chromophore. As expected, no Cotton effect was observed from the achiral oligopeptide alone, but addition of Boc-d-proline effected a CD response at 320 nm, indicating that the biphenyl groups near the C terminus find themselves in a locally chiral environment, despite being over 3 nm away from the binding event providing the chiral input. Chiral information can thus be read at the N terminus of a 3_10_-helical oligopeptide, propagate down the length of the helix, and be reported at the C terminus.

Our own group have developed a variety of methods for switching helicity in chemically robust Aib oligomers, with the induced screw-sense preference quantified by ^13^C NMR spectroscopy using isotopically enriched residues located remote from the informational input. Screw-sense switching results from diverse signals including binding chiral 1,2-diols,^92^ enantiospecific Mitsunobu reactions,^93^ and association of chiral acids with basic N-terminal residues.^94^ These examples show that a local, switchable conformational signal can induce a remote conformational response, and in a sense are conceptual models of biological communication devices such as GPCRs. However, two key differences remain: (a) biological communication devices pass information between compartments, through membranes; and (b) the ‘output’ of the communication is a change in (bio)chemical function. In order to advance the field of molecular communication devices, artificial systems must likewise be able to perform remote function as a result of informational input. However, to perform function as a result of screw-sense induction, it is crucial to understand the processes underlying the communication event and therefore how the information can be amplified.

Helical foldamers occupy a vast space of chemical architectures and can be used to arrange specific chemical functionality in a precise manner,^47,95^ and provide valuable devices for the control of shape, function and communication of information. Important examples of molecules that can change shape and therefore function in response to a stimulus include the modulation or induction of helicity in oligo(azobenzene)s,^96,97^ 2,6-bis(*N*-imidazolidin-2-onyl)pyridines,^98^ and aromatic oligoamide foldamers.^99^

Effective communication over long distances requires a conformational signal to persist as it propagates through the oligomer. This effect been explored in detail in 3_10_-helical Aib foldamers, in which the decay of screw-sense preference with distance from a chiral influence turns out to be solvent-dependent.^100^ We investigated the origin of the signal decay and proposed it was caused by the stochastic intrusion of ‘tendril perversions’, where breaks in hydrogen bonding occur randomly along the length of the helix and result in a helical reversal at that point.^101^

**Fig. 11:**
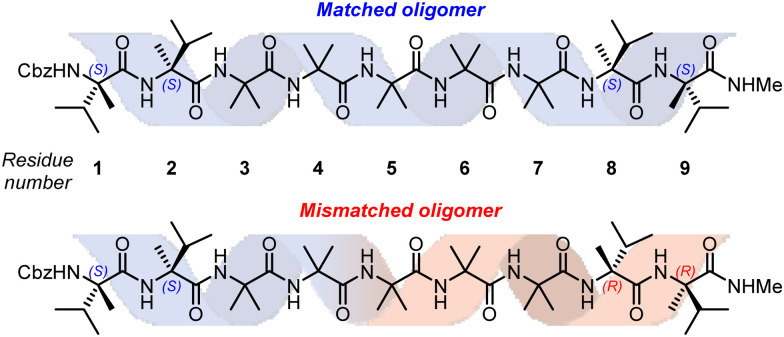
Substrates used for the investigation into tendril perversions in Aib foldamers.

In order to study this phenomenon in Aib helices, a set of Aib foldamers were terminated at each end with two α-methylvaline residues. These residues induced at one end a right-handed helix, and at the other a left-handed helix, enforcing a tendril perversion somewhere in the chain (Fig. 11). For comparison, foldamers with both screw-sense mismatched and screw-sense matched termini were synthesised.

**Fig. 12:**
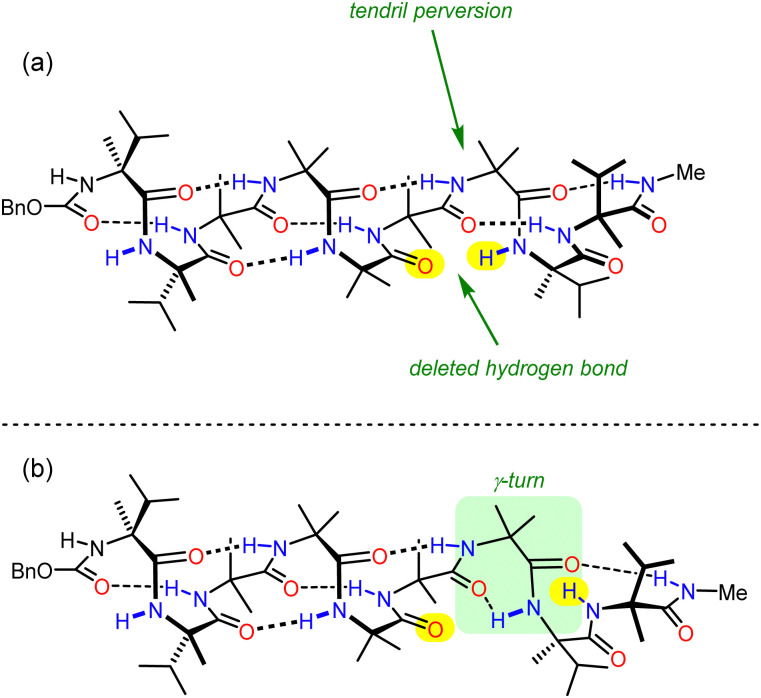
(a) Tendril perversions and (b) γ-turns in Aib foldamers.

X-Ray crystallographic studies showed a *P* screw sense along the whole length of the ‘matched’ oligomer. However, the ‘mismatched’ oligomer contained discrete *P*-helical and *M*-helical segments. A *P* helix propagated from the N terminus to the third Aib residue (residue 5), with a ‘tendril perversion’ at the fourth Aib residue, where the amide proton hydrogen bonded to a *P* helix, and carbonyl oxygen hydrogen bonded to an *M* helix (Fig. 12a), which continued to the C-terminal d-α-methylvaline. These findings were supported by NMR studies on the diastereotopic pairs of methyl groups distributed along the foldamer, which showed that consistently high screw-sense preference in the matched ‘oligomer’, dropping slightly in the centre of the helix, possibly owing to a small population of conformers containing two tendril perversions. In the ‘mismatched’ oligomer, helical excess was greatest (80% *P*) closest to the N terminus, decaying through zero to 30% *M* between the fourth and fifth Aib residues (residues 6 and 7). These findings were attributed to the presence of a single tendril perversion located between residues 5 and 7 (Fig. 12). The gradual decline in helical excess implies that the tendril perversion is a mobile feature migrating rapidly on the NMR timescale. An achiral amino acid with an appended biphenyl group as a local CD reporter for screw-sense preference likewise showed a location-dependent switch in screw sense preference.

3_10_ helices form from repeated β-turns, with hydrogen bonds between residues *i* and *i* + 3. A less prevalent turn is the γ-turn, which involves the formation of a hydrogen bond between residues *i* and *i* + 2: computational studies concluded that a γ-turn could allow the mismatched Aib oligomer to accommodate the tendril perversion. As expected, the highest population of these γ-turns was found between the centre and the C terminus. γ-Turn conformations were also more populated when simulated in more polar solvents, consistent with the empirical observation that the probability of a tendril perversion increases by 0.5% per residue in THF to 6% per residue in methanol. Knowing the mechanism by which helical fidelity in Aib-derived helical foldamers persists and decays allows communication devices to be synthesised based on and used under conditions that will moderate the decay of intramolecular communication.

**Fig. 13:**
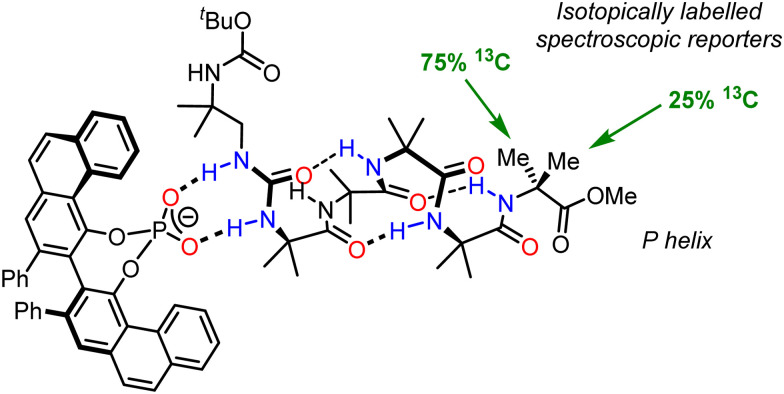
Chirality recognition and communication using a urea-appended Aib foldamer.

An important aspect of informational communication is the ability of a device to report more than one state (on/off, *S*/*R*, *P*/*M*/racemic). We have described an informational communication system incorporating an Aib oligomer that switches between conformational states by changes in pH.^94^ A key feature of the oligomer was a basic binding site which could relay information about different Brønsted acids with different p*K*_a_ values, depending on the pH of the medium. We also extended this work to an Aib foldamer that uses an *N*,*N*′-disubstituted urea as a hydrogen-bonding binding site for the recognition of geometrically complementary chiral hydrogen-bond acceptors (Fig. 13).^102^ As shown in Fig. 13, the oligomer itself is configurationally achiral (apart from isotopic labelling), so in its unbound state, the foldamer exists as a racemic mixture of helices. 1 : 1 binding of an enantioenriched phosphate anion to the urea induces a preferred screw sense, in this case *P*, which is detected by ^13^C NMR spectroscopy, using the ^13^C labelling in the terminal residue as a reporter. This example adds to the range of inputs that can be used to induce screw-sense preference, but also require some degree of selectivity: only a specific set of guests that are of sufficient hydrogen-bond-accepting ability and are of the correct geometry are able to impose a significant conformational bias on the Aib foldamer.

**Fig. 14:**
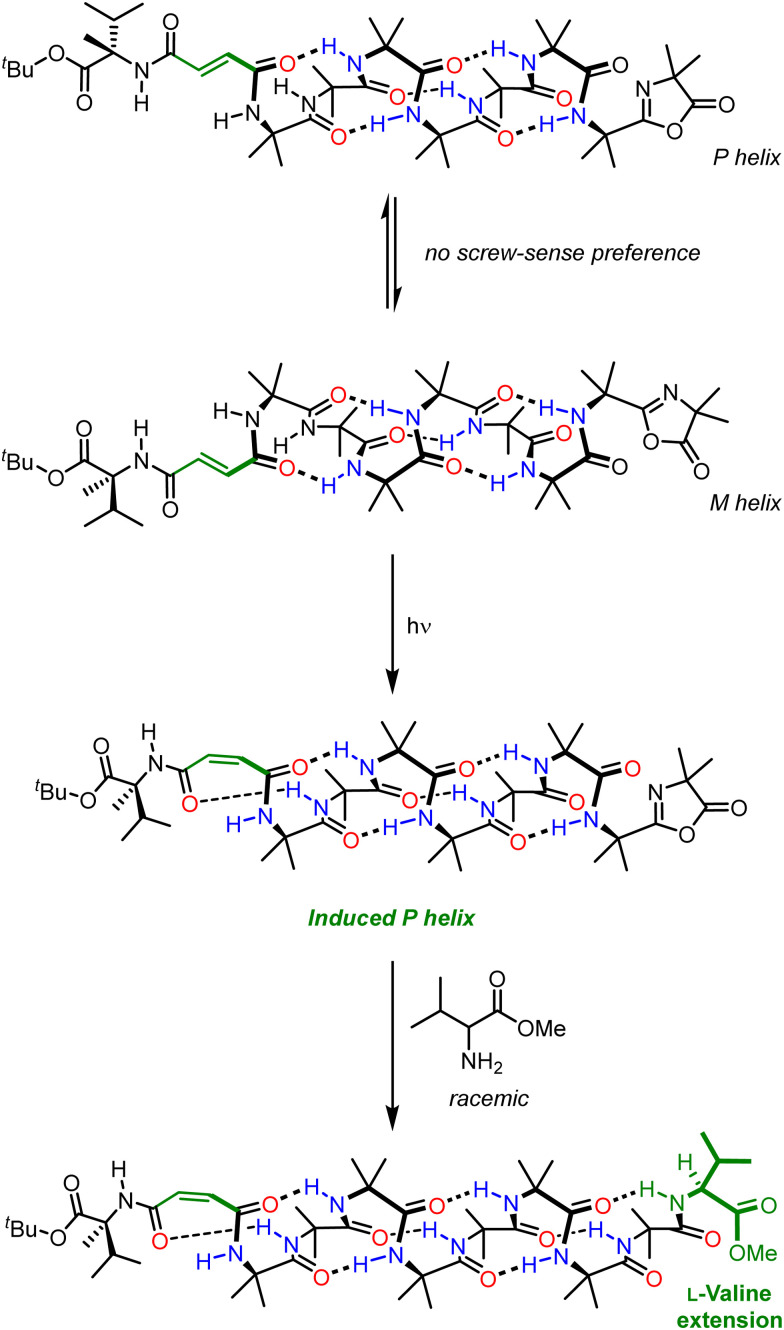
Light-modulated chiral influence in the diastereoselective chain extension of an Aib foldamer.

The need to use a chiral stimulus represents a significant limitation on the types of signal inputs that can be used to those that can exhibit some form of chirality. Many useful stimuli such as light (except circularly polarised light), heat and oxidation state are inherently achiral. Although Aib helices communicate information in the form of screw-sense preference, informational input does not necessarily need to be of a chiral nature – for example, a chiral input can be inherent to the foldamer and its influence on screw-sense preference can be modulated by an achiral stimulus as discussed previously.^91^ We investigated this phenomenon by appending a chiral fumaramide chromophore to an otherwise configurationally achiral Aib-derived foldamer.^103^ In this design, the influence of a chiral group (an l-α-methylvaline residue) on the screw-sense preference of the Aib foldamer could be turned on and off by using the different geometries of the alkene chromophore to modulate their proximity (Fig. 14). In the *E* geometry, the l-α-methylvaline is too far from the Aib oligomer for stereochemical information to be transmitted, and there was no strong bias to screw-sense preference of the Aib oligomer. Irradiation of the fumaramide isomerised it to the corresponding maleamide (*Z*), bringing the l-α-methylvaline closer to the Aib oligomer, meaning it was able to influence the screw-sense preference of the helix. Forming a reactive oxazolone on the opposite end of the helix allowed a light-promoted diastereoselective chain extension: with the (*E*) fumaramide, chain extension by the methyl ester of racemic valine resulted in a 50 : 50 mixture of diastereomers, but with the (*Z*) maleamide a diastereoselective reaction resulted in the preferential incorporation of l-valine (74 : 26 dr). This photoswitch serves as an example of screw-sense induction where a chiral influence inherent to the foldamer is modulated by light – an achiral stimulus. This development in intramolecular communication in Aib foldamers represents a significant advance as it greatly expands the range of stimuli that can be used for informational communication. It is also worth noting that the output in this example is a kinetically controlled reaction, in which selectivity may depend rather subtly on the populations and relative rate of reaction of the two screw senses, along with the rate of their interconversion in accordance with the Curtin-Hammett principle.^104^

The approach discussed here is general among communication devices, in which an appropriate receiver adopts a stimulus-dependent state and communicates that state to the channel. In this example, light serves as the stimulus and the chiral fumaramide/maleamide receiver converts the light signal into screw-sense induction.

## Screw-sense switching in the membrane phase

With repeating geminal dimethyl substituents directed towards the exterior of the 3_10_ helix, Aib foldamers can be characterised as a hydrophobic tube surrounding the twisting polar chains of hydrogen bonds within.^105^ As a consequence, both Aib foldamers and their parent peptaibols dissolve readily into phospholipid membranes. This has allowed them to function as communication devices within the membrane phase, allowing chiral information to be communicated from the exterior to the interior of a membrane-bound vesicle or cell.

Raman Optical Activity (ROA) and Vibrational Circular Dichroism (VCD) analysis was carried out in both chloroform and in dioleoylphosphatidylcholine (DOPC)-derived phospholipid bilayer vesicles.^106^ The polarity of chloroform is similar to that experienced in a phospholipid bilayer, so these studies reveal the influence of the membrane environment on the conformational preference of the foldamers. VCD spectra indicated that the relevant 3_10_ helix VCD signals in the amide I region became more intense in the vesicles for a Cbz-protected l-phenylalanine-capped Aib tetramer, suggesting that the bilayer environment either increases foldamer rigidity or increases the proportion of 3_10_-helical conformers. The enantiomeric d-phenylalanine-capped Aib tetramer gave a spectrum of the opposite sign, but with some significant differences in the amide I region, indicating that the enantioenriched environment of the phospholipid bilayer has a weak but measurable effect on the screw-sense preference of the 3_10_ helix. Additionally, in both enantiomers, several new bands appeared in the amide I region, corresponding to some α-helical character and different forms of bends and turns. Comparison of the signs of the spectra indicate that the screw-sense preference induced in the solution phase is the same as that induced in bilayers. Crucially, this study shows that 3_10_-helical content is persistent in phospholipid bilayers, meaning that Aib-derived foldamers could serve as transmembrane communication devices that can respond to external stimuli.^106^

**Fig. 15:**
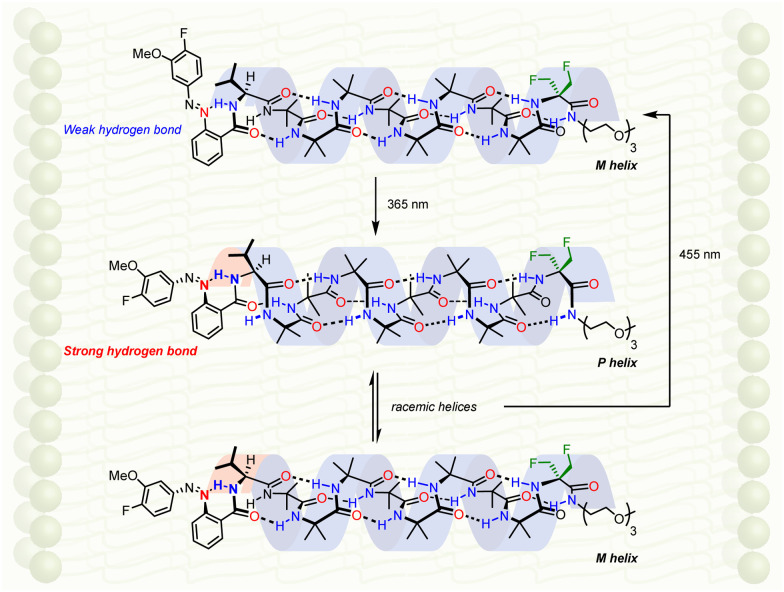
Light-modulated influence of chirality in an Aib foldamer in DOPC-derived vesicles.

Screw-sense modulation as a result of a configurational change induced by light was translated into the membrane phase by ligation of a chiral photosensitive moiety to the N terminus of an Aib_8_ foldamer (Fig. 15).^107^ An enantioenriched l-valine residue biases the helical equilibrium towards a right-handed helix, but this influence may be modulated by a photoresponsive azobenzenecarboxamide attached to the l-valine. In deuterated methanol, deuterated chloroform and DOPC-derived membranes, a C-terminal geminal di(fluoromethyl) group was suitable reporter of screw-sense preference: both solution and solid-state ^19^F NMR spectroscopy gave well resolved spectra that could be used to analyse screw-sense preference without the overlapping signals that would necessarily arise from other nuclei. A fluorine-substituted azobenzene group allowed the same ^19^F spectrum to report on azobenzene geometry.

In the azobenzene *E* configuration, the lone pair of electrons on the proximal diazo nitrogen forms a hydrogen bond with the amide proton of the l-valine. This hydrogen bond was weak, and therefore the l-valine's influence on the screw sense of the foldamer was largely unaffected, giving rise to a left-handed preference in the bilayer. Irradiation with light at 365 nm converted the azobenzene mostly to the corresponding *Z* configuration (18 : 82 *E *:* Z*). As a result, to mitigate the steric encumbrance associated with coplanarity of the arenes, the diazo group is distorted, making the diazo nitrogen more basic, strengthening the hydrogen bond to the adjacent l-valine amide proton, and weakening the l-valine's influence on the Aib foldamer. A racemic mixture of helical conformations results (Fig. 15). Further irradiation at 455 nm restores the original screw-sense preference.

The ability of the foldamer to respond to light is reminiscent of the function of the vision proteins such as rhodopsin. In rhodopsin, a configurational change in a chromophore is translated into a change in the relative populations of alternative conformations of a membrane-embedded oligomer (here, a foldamer; in rhodopsin, the protein), leading to a detectable remote effect (here, an NMR spectroscopic response; the release of transducin in rhodopsin).

Other biomimetic functions in a membrane requires intermolecular interactions that are sufficiently strong to persist even in an aqueous environment. In order to strengthen these interactions, we made use of a metal cation bound within a bis(2-quinolylmethyl)(2-pyridylmethyl)amine (BQPA) pocket, that was also capable of binding anionic ligands such as chiral carboxylates. We used this anion-responsive binding site to transmit chiral information in a DOPC-derived vesicle in the form of an induced screw-sense preference.^108^ Conceptually, the design constitutes a mimic of the GPCR communication mechanism: binding of a ligand resulted in a conformational change which propagated within a lipid bilayer.

**Fig. 16:**
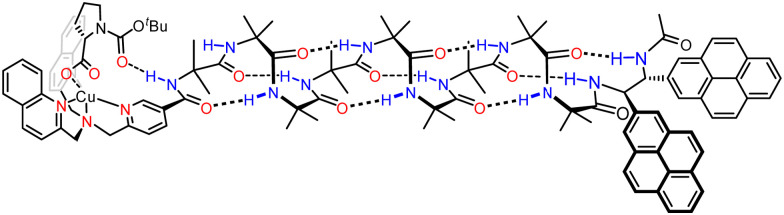
Screw-sense induction using enantioenriched carboxylate ligands for a Cu(ii) cofactor.

The detailed design of the receptor mimic is shown in Fig. 16. The BQPA pocket was appended at the N terminus of an Aib_8_ foldamer and bound to a Cu(ii) ‘cofactor’, which itself interacts with enantioenriched amino acid-derived carboxylate ligands. With NMR spectroscopy methods being unsuitable for use in vesicles, a chiral bis(pyrene) fluorophore was developed and used to terminate the Aib_8_ foldamer. Being chiral, the fluorophore interacted differently with the right- and left-handed screw senses of the Aib helix, meaning that fluorescent responses were dependent on the screw-sense preference of the helix. This bis(pyrene) reporter was carefully selected to ensure that the interaction between the chiral bis(pyrene) and the foldamer was too weak to bias screw-sense preference, but strong enough to respond to a conformational change induced from the N terminus by reorientating the two pyrene rings.^109^ In the absence of ligand for the Cu(ii), left- and right-handed helices were populated approximately equally.

**Fig. 17:**
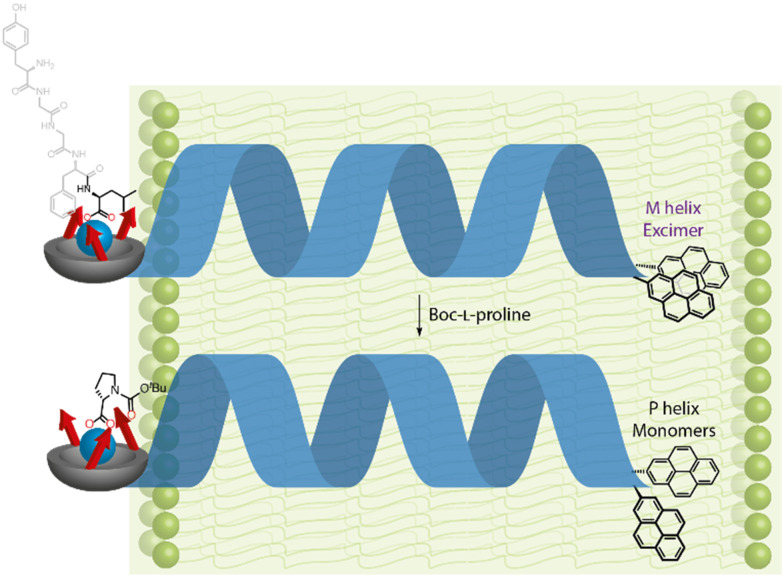
Reversal of screw-sense preference in DOPC-derived vesicles using carboxylate ligands.

With the Cu(ii)-bound foldamer embedded in the DOPC phospholipid bilayer of unilamellar vesicles, the ratio of excimer (*E*) to monomeric (*M*) contributions to the fluorescence spectrum could be perturbed by binding of carboxylate ligands. Boc-l-proline was bound to the Cu(ii)-BQPA in the bilayer, inducing a right-handed helix, causing the two pyrenes to move away from each other and decreasing the *E*/*M* ratio (Fig. 17). With Boc-d-proline, a left-handed helix was induced, bringing the two pyrene units closer together, promoting excimer emission and increasing the *E*/*M* ratio. The GPCR mimic also responded to the natural opioid peptide neurotransmitter, leu-enkephalin, which is known to bind to μ- and δ-opioid GPCRs. By determining the half-maximal effective concentration values of the various carboxylates by fitting to *E*/*M* ratio changes, it was shown that leu-enkephalin induced a left-handed helix as strongly as Boc-d-proline. This meant that a leu-enkephalin ‘agonist’ and a Boc-l-proline ‘antagonist’ could competitively bind to the Cu(ii)-BQPA, inducing reversible and opposite fluorescent responses depending on the relative concentrations of leu-enkephalin and Boc-l-proline.

This functional GPCR mimic thus binds chiral ligands at a binding site incorporating a Cu(ii) cofactor, and relays the chiral information through the bilayer to deliver a remote fluorescence response over 2.6 nm away, as a result of conformational communication through the Aib foldamer.

The use of Aib foldamers for communicating information has the potential for further exploitation in biological settings, and more recent advances in the synthesis of Aib-containing oligomers by automated solid-phase peptide synthesis facilitates these applications.^110,111^ Future advances could see the development of Aib foldamers which modulate binding affinity or p*K*_a_ of C-terminal functions, with the potential for induced release or uptake of molecules within a cell in response to an external stimulus.

## Screw-sense switching in helical oligoureas

The formation of a helix requires a pattern of attractive interactions between monomers, adjacent or otherwise. Hydrogen bonding is a prevalent driving force for the formation of helices, with amides commonly being the functional group of choice due to their ability to act as both strong hydrogen-bond donors and acceptors. Amides and amide oligomers are however inherently directional: oligoamides will always possess an overall dipole moment due to their constitutionally defined N and C terminus.

Ureas are also strong hydrogen-bond donors and acceptors, but have the added feature of being able to donate two hydrogen bonds, with consequences for the strength and geometry of the hydrogen bonds they form.^112^ This can result in the formation of helices unlike those encountered in natural oligomers.^113,114^ Intriguingly, the constitutional symmetry associated with urea functional group means that the overall dipole moment of an oligourea is not necessarily constitutionally defined, and can therefore potentially be influenced by external stimuli.

**Fig. 18:**
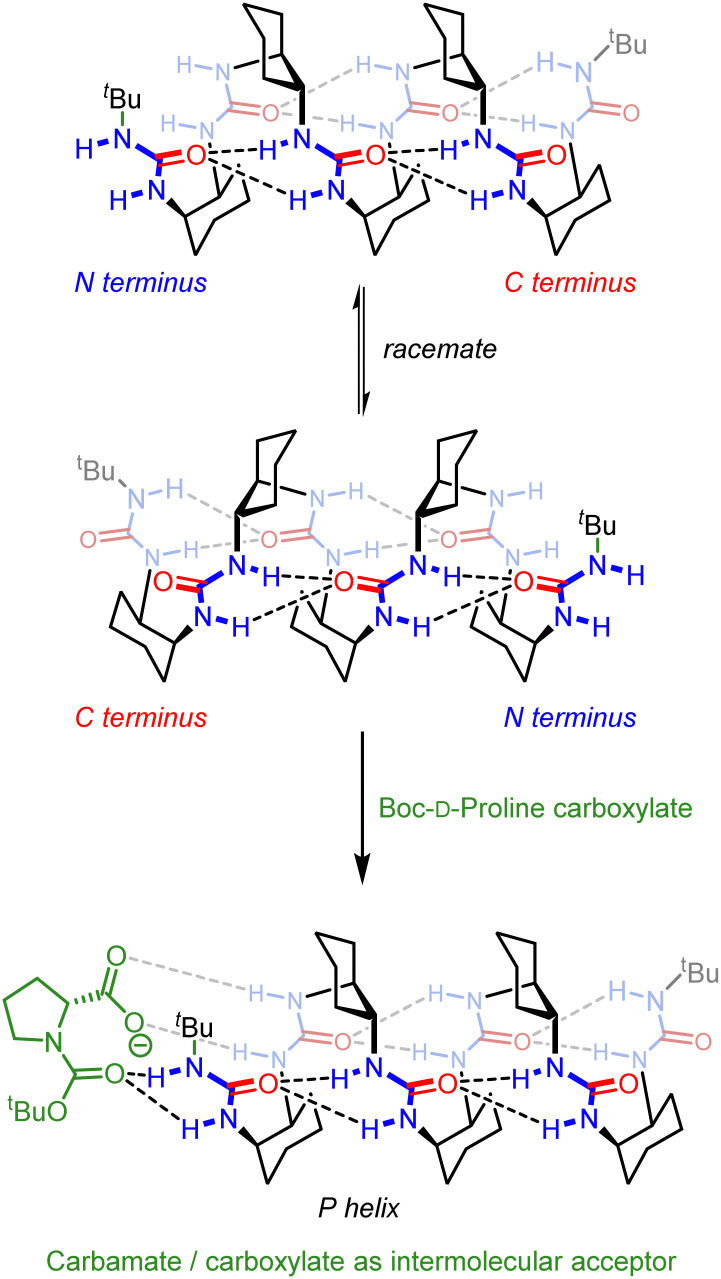
Induction of hydrogen-bond directionality and screw-sense preference in 2.5_12/14_-helical oligourea foldamers. 2.0 Helix depicted for clarity.

Guichard and co-workers pioneered the development of urea-derived helical foldamers, which have found use as asymmetric catalysts, peptidomimetics and as bactericides.^54,115,116^ In collaboration with Guichard, we synthesised oligoureas derived from the *meso* monomer *cis*-cyclohexane-1,2-diamine.^117^ X-Ray crystallographic studies showed that in the solid state, these oligoureas existed as 2.5_12/14_ helices with hydrogen bonds between the ureido carbonyl groups and the two ureido protons of the monomer two units ahead of it (Fig. 18). As these helices were comprised of *meso*, and therefore achiral, monomers, left- and right-handed screw senses were equally populated when the termini of the helices were identical. Variable-temperature NMR spectroscopy showed that screw-sense inversion was slow on the NMR timescale, with a barrier Δ*G*^‡^_298 K_ = 70 kJ mol^−1^ in chloroform. This value lowered to 66 kJ mol^−1^ in methanol due to competitive hydrogen-bonding interactions. Helix inversion necessitates cyclohexane ring-flipping, but the ring flip typically has a much lower barrier (<50 kJ mol^−1^), so this high barrier was attributed to rate-limiting hydrogen bond reorganisation. Interestingly, helical inversion is accompanied by a hydrogen-bond directionality reversal and therefore a switch of the N (*i.e.* hydrogen-bond donating) and C (hydrogen-bond accepting) termini.

**Fig. 19:**
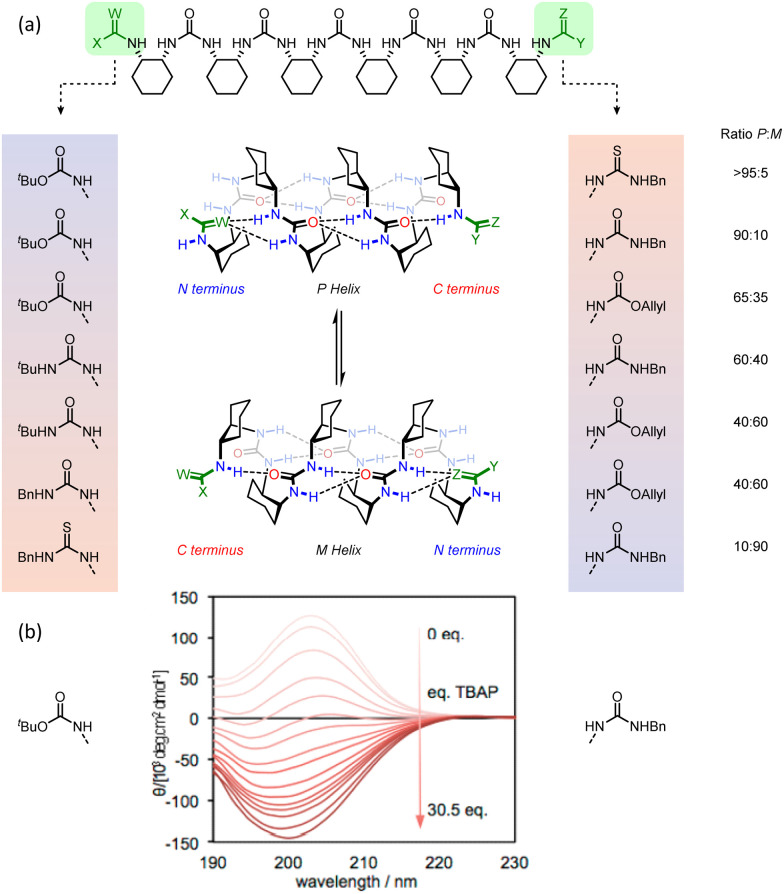
(a) 2.5_12/14_-helical oligourea foldamers as torsion balances for hydrogen-bonding strength. (b) Screw-sense reversal and hydrogen-bond-directionality reversal upon addition of tetrabutylammonium phosphate. The end groups of the foldamer used in the titration are displayed on either side of the CD traces. Reprinted (adapted) with permission from ref. 118. Copyright 2020 American Chemical Society.

The intimate coupling between hydrogen-bond directionality and screw sense means that desymmetrisation of the helix using different terminating groups gives rise to a screw-sense preference.^118^ For example, in the pentaurea, functionalisation of one terminus with *tert*-butoxycarbonyl and the other with allyloxycarbonyl gives rise to a 2.1 : 1 ratio of *P *:* M* helices in CD_3_CN at 25 °C, as shown by ^1^H NMR and CD spectroscopy (Fig. 19).

This bias arises from the differing hydrogen-bonding properties of *tert*-butyl and allyl carbamates, with the (more electron-rich) *tert*-butyl carbamate preferring the N terminus and the allyl carbamate the C terminus in the major conformer. Modifications of the termini allowed modulation of the helical excess in these oligourea foldamers, with competitive hydrogen-bond donation and acceptance of the termini making the helices torsion balances for hydrogen-bond strength. Thioureas are much stronger hydrogen-bond donors than their homologous ureas.^119^ With an *N*-benzyl thiourea at one terminus and an *N*-benzyl urea at the other, a 10 : 90 ratio of *P *:* M* helices was formed. Replacing the urea with a carbamate, a poorer hydrogen-bond acceptor, inverted the ratio to >95 : 5 *P *:* M*.

Desymmetrisation of the helix was also achieved through intermolecular interactions. *N*-Boc-prolinate is a multiple hydrogen-bond acceptor through the carbamate and carboxylate, and is of a suitable geometry to form four hydrogen bonds with the first two ureas of a *meso* oligourea helix (Fig. 18). Titrating a *meso* heptaurea with Boc-d-proline induced a right-handed screw-sense preference with the ligand binding at the N terminus of the urea helix.

An achiral ligand tetrabutylammonium phosphate induced a conformational switch in one of the thiourea-terminated foldamers, which switched hydrogen-bond directionality in order for the thiourea to hydrogen-bond to the phosphate. Hydrogen-bond-directionality reversal was also associated with a screw-sense reversal as shown by the inversion of the CD spectrum upon addition of phosphate (Fig. 19b).

Oligoureas and oligoamides may be interfaced: the sensitivity of the oligourea foldamers to hydrogen-bond directionality and that of Aib foldamers to chirality was explored by synthesising an oligopeptide/oligourea hybrid.^120^ Central chirality in the oligourea imposed screw-sense preference onto the configurationally achiral all-Aib oligopeptide domain, while the C-terminus of the oligopeptide domain enforced the oligourea's N terminus to reside at the interdomain interface. This constituted a cooperative helical foldamer with two distinct helical domains with correlated screw-sense preference and hydrogen-bond directionality.

**Fig. 20:**
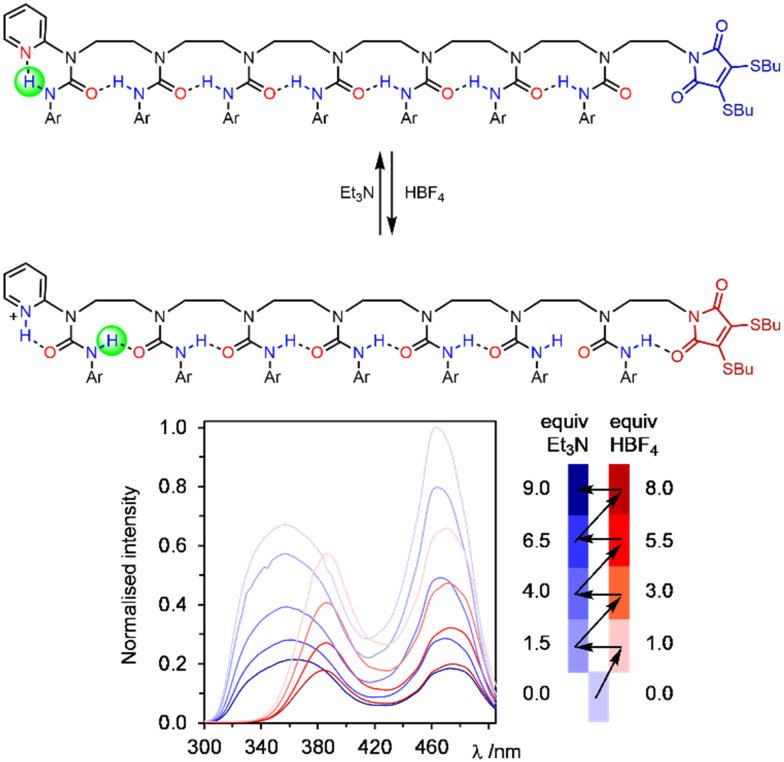
Oligourea foldamers that communicate a terminal pyridine's protonation state as hydrogen-bond polarity information without using chirality. Adapted from open access journal ref. 121.

The ability to induce hydrogen-bond directionality provides an alternative mechanism for communication which does not require helices at all. We used this concept to synthesise oligoureas devoid of chirality that read information in the form of the protonation state of acidic or basic transmitters, and communicated it down an oligourea chain, remotely controlling the conformation of a urea seven units away and giving a local spectroscopic response (Fig. 20).^121^ This work was extended to remotely modulating the affinity of a urea binding site to a phosphine oxide ligand, this time communicating information over five urea units to confer a fivefold increase in binding affinity.^122^ In both of these cases, the protonation state of the transmitter could be switched back and forth numerous times, each time being communicated down the length of the oligourea and remotely carrying out its spectroscopic or chemical function.

## Conclusions

Synthetic chemistry has classically addressed the molecular challenges presented by biological structure. But in its coming to maturity, synthesis has also enabled the development of families of dynamic molecules that also aim to mimic biological function, among them arguably the most important facet of nature – the ability to process information. From a relatively small library of prebiotic molecules, nature has evolved to make exceedingly complex architectures to perform any task an organism may need. Synthetic chemistry has alternative resources – the power of the periodic table and the ingenuity of chemists – and this informed design is aiming to develop molecules that can store, communicate and process information in a similar way. Dynamic foldamers have proved to be effective in information storage, communication and processing in simulated biological settings and in response to a range of physicochemical stimuli. Neither screw-sense switching nor hydrogen-bond directionality switching are employed in biological contexts, and this ability to expand biological concepts to new structural contexts could see new ways of generating chemical function by processing information, including the development of hydrogen-bond directionality as a communication mechanism.

## Abbreviations

GPCR: G-Protein coupled receptor
Aib: α-Aminoisobutyric acid
THF: Tetrahydrofuran
PQX: Poly(quinoxaline-2,3-diyl)
SANS: Amall-angle neutron scattering
CD: Circular dichroism
TCE: 1,1,2,2-Tetrachloroethane
HPLC: High-performance liquid chromatography
DMSO: Dimethylsulfoxide
NMR: Nuclear magnetic resonance
Boc: *tert*-Butyloxycarbonyl
dr: Diastereoisomeric ratio
ROA: Raman optical activity
VCD: Vibrational circular dichroism
DOPC: Dioleoylphosphatidylcholine
BQPA: Bis(2-quinolylmethyl)(2-pyridylmethyl)amine
TBAP: Tetrabutylammonium phosphate

## Conflicts of interest

The authors declare no conflict of interest.

## Supplementary Material
